# Incorporating indel information into phylogeny estimation for rapidly emerging pathogens

**DOI:** 10.1186/1471-2148-7-40

**Published:** 2007-03-14

**Authors:** Benjamin D Redelings, Marc A Suchard

**Affiliations:** 1 Bioinformatics Research Center, North Carolina State University, Raleigh, NC 27606, USA; 2 Department of Biomathematics, David Geffen School of Medicine at UCLA, Los Angeles, CA 90095, USA; 3 Department of Human Genetics, David Geffen School of Medicine at UCLA, Los Angeles, CA 90095, USA; 4 Department of Biostatistics, UCLA Schoold of Public Health, Los Angeles, CA 90095, USA

## Abstract

**Background:**

Phylogenies of rapidly evolving pathogens can be difficult to resolve because of the small number of substitutions that accumulate in the short times since divergence. To improve resolution of such phylogenies we propose using insertion and deletion (indel) information in addition to substitution information. We accomplish this through joint estimation of alignment and phylogeny in a Bayesian framework, drawing inference using Markov chain Monte Carlo. Joint estimation of alignment and phylogeny sidesteps biases that stem from conditioning on a single alignment by taking into account the ensemble of near-optimal alignments.

**Results:**

We introduce a novel Markov chain transition kernel that improves computational efficiency by proposing non-local topology rearrangements and by block sampling alignment and topology parameters. In addition, we extend our previous indel model to increase biological realism by placing indels preferentially on longer branches. We demonstrate the ability of indel information to increase phylogenetic resolution in examples drawn from within-host viral sequence samples. We also demonstrate the importance of taking alignment uncertainty into account when using such information. Finally, we show that codon-based substitution models can significantly affect alignment quality and phylogenetic inference by unrealistically forcing indels to begin and end between codons.

**Conclusion:**

These results indicate that indel information can improve phylogenetic resolution of recently diverged pathogens and that alignment uncertainty should be considered in such analyses.

## Background

Reconstructing viral phylogenies is important for determining the parent stock of newly emerging strains [1], as well as for understanding how viruses evolve over time, both within a single host and at the population level [2]. Viral phylogenies are commonly inferred from aligned molecular sequence data, using the information available in substitutions shared by descent [3-6]. Short time-scales dominate in the development of rapidly emerging disease strains, such that the number of observed substitutions between sequences can be too low to yield well-resolved phylogenies. Thus, to increase phylogenetic resolution for such disease strains we seek to make use of a wider class of phylogenetic information.

Insertions and deletions (indels) are a promising category of molecular sequence information that is largely ignored in phylogenetic reconstruction. Researchers commonly remove gaps from molecular sequences alignments by coding them as missing data or by throwing out columns that contain gaps [3-6]. Indels may be useful to resolve deep branches in the Tree of Life that are difficult to resolve using information in shared substitutions [7,8]. At the other extreme, on which we focus here, indels can help to resolve phylogenies in situations where the number of nucleotide substitutions is inadequate. For example, indels in non-coding chloroplast DNA have been helpful in resolving the branching order of recent plant radiations [9,10]. The rate of indel events in these regions approaches or surpasses the rate of substitution, making indels too important to ignore [10]. Several species of viruses are also known to accumulate indels, sometimes at a high rate. Cheyner et al. [11] note that indel rates are higher than substitution rates in hyper-variable regions of Simian Immunodeficiency Virus (SIV) and Human Immunodeficiency Virus (HIV). Other viruses also experience indels on short time-scales. Hepatitis B virus (HBV) accumulates deletions in the core/pre-core region during the course of infection [12], while Equine Infectious Anemia Virus accumulates insertions [13]. Three deletion variants of Severe Acute Respiratory Syndrome (SARS) appeared during the beginning of the SARS outbreak in China [14]. Influenza B viruses accumulate indels over several decades [15]. We note that these viruses are all RNA viruses, with the exception of HBV. Although HBV is a DNA virus, it reverse transcribes its DNA genome from an RNA intermediate.

Redelings and Suchard (2005) describe a statistical method of incorporating indel information into phylogeny estimation. This method uses a joint reconstruction framework that simultaneously infers the alignment, tree, and insertion/deletion rates. Estimation proceeds through Markov chain Monte Carlo (MCMC) within a Bayesian framework and naturally accounts for uncertainty in alignments, phylogenies, and other parameters through posterior probabilities. Unlike sensitivity analysis [16,17], this approach takes into account uncertainty resulting from the myriad of near-optimal alignments. This approach involves averaging over unobserved quantities such as the alignment and interal node states, which can lead to improved estimates [18]. This is different from other approaches which iteratively optimize a heuristically chosen cost function until no improvement is seen [19,20]. Joint estimation of alignment and phylogeny sidesteps bias that results from conditioning on a single alignment estimate [21,18], bias which may be exaggerated when indel information is inappropriately used.

This method is based on a probabilistic model of sequence evolution that contains insertion and deletion events as well as substitution events. Heuristic "costs" for opening and extending gaps are replaced by the insertion/deletion rate and the mean indel length respectively, which are biologically interpretable parameters and can be estimated from the data without circularity [22,23]. Gaps are not treated as a fifth character state, since this overweights the evidence of shared indels by treating an indel of multiple residues as multiple shared indels [3]. Instead, the indel process is separate and independent of the substitution process, and allows indels of several residues simultaneously. In addition, because alignments represent positional homology, the indel process does not allow a newly inserted character to be aligned to a previously deleted character.

We introduce a new indel model to remedy a shortcoming of the Redelings and Suchard (RS05) model. Unlike the TKF1 [22] and TKF2 [23] indel models that are not reversible on pairwise alignments, the reversible RS05 model does not make use of branch length information in the indel process and therefore does not place indels preferentially on longer branches. In order to increase biological realism, we describe an extended indel model that is able to incorporate branch length information. In doing so we overcome a substantial theoretical difficulty in using reversible indel models during phylogenetic reconstruction.

We further enhance the estimation method of Redelings and Suchard [24] by introducing a novel MCMC transition kernel to improve mixing among topologies. This transition kernel is based on the subtree-prune-and-regraft (SPR) operator but is modified to partially sample the alignment along with the tree. Block sampling improves mixing efficiency because topologies and alignments are highly inter-correlated.

We introduce codon models [25] into joint estimation. Codon models are often used in both Bayesian and likelihood-based phylogeny estimation because they naturally allow different rates at the third codon position, but we are not aware of any work using codon models in joint estimation. We note that codon models implicitly alter the indel process as well as the substitution process by forcing indels to begin and end between codons. This constraint may not be biologically realistic and would result in misaligned nucleotides when indels are not in phase with the reading frame. Such misalignment can artificially inflate the number of inferred substitutions. When the total number of substitutions is small, this may significantly alter the model fit or introduce bias. We compare nucleotide and codon indel models to see if these effects are significant.

We analyze data sets from SIV and HIV. The SIV data set consists of a short section of the envelope (*env*) gene from 9 within-host strains. To see if indel information improves phylogenetic resolution we compare the number of bi-partitions that are supported under the joint model and the traditional sequential approach, in which topology reconstruction assumes a previously determined alignment. We also assess the importance of alignment ambiguity by assessing the sensitivity of phylogeny estimation to fixed alignments under both the traditional and joint models. The HIV data set consists of about 600 nucleotides from the *env *gene from 27 within-host strains. We compare the number of bi-partitions supported under the sequential and joint models to assess the importance of indel information. We also compare nucleotide and codon models to see if the assumption of unbreakable codons significantly decreases model fit or influences phylogeny estimates.

In summary, we seek to improve the power to infer clades in rapidly emerging taxa by making use of indel information in a statistically rigorous manner. We also seek to determine whether indels can actually resolve extremely short branches with few substitutions. To accomplish these goals, we introduce an improved statistical model of the insertion-deletion process to improve the accuracy of the inference, and describe a novel MCMC transition kernel to improve the speed of the inference. Once our statistical framework is in place, we then demonstrate that indel information can help to detect previously undetected bi-partitions in two real data examples from RNA viruses. While analyzing these data, we note that alignment ambiguity may significantly affect phylogeny inference. We note that codon-based alignments can unrealistically shift indels to avoid breaking codons, and we develop the necessary statistical machinery to demonstrate that this can substantially affect phylogeny estimates.

## Results

### Models and Algorithms

We introduce a time-dependent reversible indel process to the probabilistic framework for joint estimation of alignment and phylogeny of Redelings and Suchard [24]. Time-dependence enables us to place indels preferentially on longer branches of the tree, producing a more realistic description of the evolutionary process. Further, we also introduce a novel MCMC transition kernel to increase topology mixing so that we can estimate phylogenies and alignments containing increasingly more taxa.

#### Stochastic Model

We review the salient features of the RS05 model here and propose the necessary extensions for a time-dependant indel process. Our model starts with data **Y**, where **Y **is a collection of unaligned molecular sequences **Y**_*i *_for *i *= 1, ..., *n *taxa. Each molecular sequence **Y**_*i *_is a collection of letters of length |**Y**_*i*_|. We characterize the stochastic model that describes how the sequences in **Y **diverged from a common ancestor in terms of a number of unknown but estimable parameters. These parameters include a multiple alignment **A **that specifies the positional homology between the sequences **Y**, an evolutionary tree (*τ*, **T**) where *τ *is an unrooted bifurcating tree topology and **T **= (*t*_1_, ..., *t*_2*N *-3_) is a vector of branch lengths along the edges in *τ*, and vectors **Θ **and **Λ **are parameters that characterize the letter substitution and indel processes respectively. Alignment **A **includes Felsenstein wildcard sequences of random lengths at the internal nodes of *τ*. Thus, **A **also depicts the complete indel history among the sequences in **Y**. We scale branch lengths in terms of expected number of substitutions per site.

In contrast to traditional methods of phylogeny estimation that arbitrarily fix the alignment, we treat the alignment **A **as a random variable, leading to the probability expression

P(**Y**, **A**, *τ*, **T**, **Θ**, **Λ**) = P(**Y**|**A**, *τ*, **T**, **Θ**) × P(**A**|*τ*, **T**, **Λ**) × P(*τ*, **T**) × P(**Θ**) × P(**Λ**).     (1)

The substitution likelihood P(**Y**|**A**, *τ*, **T**, **Θ**) and the priors P(*τ*, **T**) and P(**Θ**) occur in traditional Bayesian models that fix the alignment. However, the alignment prior P(**A**|*τ*, **T**, **Λ**) and the prior on indel process parameters P(**Λ**) are novel in the joint model, allowing for estimation and a natural way to handle uncertainty in **A**.

##### Substitution Model

To model the substitution process that specifies P(**Y**|**A**, *τ*, **T**, **Θ**), we assume that substitutions in each column of **A **occur independently and follow a continuous-time Markov chain (CTMC) process [26]. Under this process, letters at the root of the tree arise according to some distribution *π*. Evolution then occurs independently along each branch of *τ *with rate matrix **Q**. We restrict ourselves to reversible Markov chains and use *π *as the equilibrium distribution of **Q**. This makes the position of the root unidentifiable and so we use unrooted trees throughout this paper.

CTMC models are in common usage for letters from nucleotide-, codon-, and amino acid-based alphabets. In contrast to nucleotide-based CTMC models, codon-based models group the three nucleotides in a codon into a single letter. Given the small number of substitutions that occur during the emergence of rapidly evolving pathogens, codon-based models are preferred over amino-acid based models because they do not discard synonymous substitutions. Codon-based models can also improve model efficiency over nucleotide-based models because the codon-based models can include non-independent nucleotide frequencies and rule out missense mutations [25]. Codon-based models may also improve the accuracy of estimation by allowing the third-codon position to evolve at a higher rate. However, when the number of observed substitutions is low it may not be possible to estimate the non-synonymous to synonymous rate ratio *ω*, requiring researchers to fix *ω *to a previously estimated value.

Importantly, we note that codon-based models also affect the indel process by forbidding frameshift mutations and also indels that begin or end within a codon. While the former constraint is realistic for biologically active viruses, the latter constraint may force incorrect alignments at the nucleotide level, causing up to two misaligned residues per indel. This may result in a significant bias when the total number of substitutions is small.

##### Indel Models

Redelings and Suchard [24] make the simplifying assumption that the alignment prior

P(**A**|*τ*,**T**,**Λ**) = P(**A**|*τ*, **Λ**)     (2)

is independent of branch lengths. While this assumption implies that indels are equally likely to occur on each branch regardless of length, it trivially enforces that sequence length distributions *φ *on all nodes in *τ *remain the same. This is a necessary condition for constructing a reversible evolutionary Hidden Markov model (HMM) from pair-HMMs along the branches of *τ*. Reversibility substantially decreases implementation complexity. The assumption further allows us to avoid fragment based pair-HMMs that tend to separate indels by the average indel length, which is not necessarily biologically realistic.

Here we develop an alignment prior P(**A**|*τ*, **T**, **Λ**) that explicitly depends on branch lengths but retains equivalent sequence length distributions on all nodes of the tree. We begin construction of the extended model by briefly summarizing how the original indel model is constructed from a pairwise alignment distribution *ν*. We modify this construction to build the new indel model from a parameterized distribution *ν*_*t *_on pairwise alignments that corresponds to a divergence time *t*. We then describe a new pair-HMM which serves to generate *ν*_*t*_. Finally we describe how to calculate posterior probabilities under this model.

To describe our original multiple alignment model, we begin by noting that, given a topology *τ*, the multiple alignment **A **can be decomposed into a set of pairwise alignments **A**^(*b*) ^along each branch *b *of the topology. This decomposition is possible because of the inclusion of Felsenstein wildcard sequences at the internal nodes of *τ*. Imposing an arbitrary distribution *ν *on each pairwise alignment **A**^(*b*) ^independently yields a joint distribution over **A**. However, pairwise alignments on neighboring branches are not strictly independent because they both specify the length of the random sequence at the node they share. To handle this dependence, we first choose an arbitrary internal node in *τ *as the root; this imposes an orientation on each branch. We then label the sequence in each branch alignment **A**^(*b*) ^that is closest to the root as the ancestral sequence and the other sequence as the descendant sequence. We sample the sequence length at the root from a distribution φ˜
 MathType@MTEF@5@5@+=feaafiart1ev1aaatCvAUfKttLearuWrP9MDH5MBPbIqV92AaeXatLxBI9gBaebbnrfifHhDYfgasaacH8akY=wiFfYdH8Gipec8Eeeu0xXdbba9frFj0=OqFfea0dXdd9vqai=hGuQ8kuc9pgc9s8qqaq=dirpe0xb9q8qiLsFr0=vr0=vr0dc8meaabaqaciaacaGaaeqabaqabeGadaaakeaaiiGacuWFgpGzgaacaaaa@2E7B@ and draw the pairwise alignment **A**^(*b*) ^for each branch *b *from *ν *conditional on the length of the ancestral sequence, proceeding down the tree from the root to the leaves.

We note that the pairwise alignment distribution *ν *induces a sequence length distribution on each sequence in the pair it emits. To proceed, we require that the pairwise alignment distribution *ν *be symmetric under interchange of the two sequences in the pair. This implies that there is no preferred direction of evolution between the two sequences. It also implies that the sequence length distribution for the ancestral and descendant sequences are equal; we call this common distribution *φ*. If we set the root length distribution φ˜
 MathType@MTEF@5@5@+=feaafiart1ev1aaatCvAUfKttLearuWrP9MDH5MBPbIqV92AaeXatLxBI9gBaebbnrfifHhDYfgasaacH8akY=wiFfYdH8Gipec8Eeeu0xXdbba9frFj0=OqFfea0dXdd9vqai=hGuQ8kuc9pgc9s8qqaq=dirpe0xb9q8qiLsFr0=vr0=vr0dc8meaabaqaciaacaGaaeqabaqabeGadaaakeaaiiGacuWFgpGzgaacaaaa@2E7B@ = *φ*, then we can write the multiple alignment prior as

P(A|τ,Λ)=∏b=1BPν(A(b))∏i∈Iφ(|Ai|)2,     (3)
MathType@MTEF@5@5@+=feaafiart1ev1aaatCvAUfKttLearuWrP9MDH5MBPbIqV92AaeXatLxBI9gBaebbnrfifHhDYfgasaacH8akY=wiFfYdH8Gipec8Eeeu0xXdbba9frFj0=OqFfea0dXdd9vqai=hGuQ8kuc9pgc9s8qqaq=dirpe0xb9q8qiLsFr0=vr0=vr0dc8meaabaqaciaacaGaaeqabaqabeGadaaakeaacqqGqbaucqGGOaakieqacqWFbbqqcqGG8baFiiGacqGFepaDcqGGSaaliiqacqqFBoatcqGGPaqkcqGH9aqpdaWcaaqaamaaradabaGaeeiuaa1aaSbaaSqaaiab+17aUbqabaGccqGGOaakcqWFbbqqdaahaaWcbeqaaiabcIcaOiabdkgaIjabcMcaPaaakiabcMcaPaWcbaGaemOyaiMaeyypa0JaeGymaedabaGaemOqaieaniabg+Givdaakeaadaqeqaqaaiab+z8aMjabcIcaOiabcYha8jab=feabnaaBaaaleaacqWGPbqAaeqaaOGaeiiFaWNaeiykaKYaaWbaaSqabeaacqaIYaGmaaaabaGaemyAaKMaeyicI4SaemysaKeabeqdcqGHpis1aaaakiabcYcaSiaaxMaacaWLjaWaaeWaaeaacqaIZaWmaiaawIcacaGLPaaaaaa@5B20@

where *I *represents the set of internal nodes in *τ *[24]. Note that in this expression the arbitrary root is not identifiable.

Unfortunately the parameters that characterize our original pairwise alignment distribution *ν *can not vary from branch to branch without inducing unequal length distributions. We therefore propose a new pairwise alignment prior that maintains a fixed sequence length distribution *φ *even when the indel probability varies from branch to branch. To accomplish this aim, we assume that each sequence consists of a series of unbreakable fragments, as in the TKF2 model. The fragment lengths are geometrically distributed with continuation probability *ε *and minimum length 1. The number of fragments is uniformly distributed over the non-negative integers. Following an ancestral fragment at one end of a branch, a geometric number of new fragments are inserted in the descendent with continuation probability *δ*(*t*). Each ancestral fragment survives in the descendent with probability *δ'*(*t*) = *δ*(*t*)/(1 - *δ*(*t*)). Following our previous model and the TKF models, insertions and deletions are equally likely.

**Figure 1:**
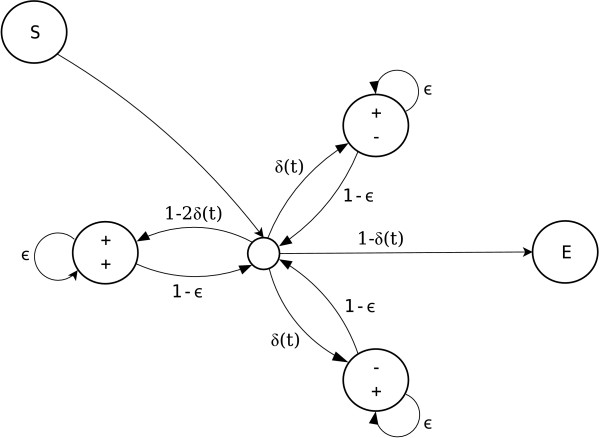
**Pair-HMM representation of the fragment-based indel model**. After the start state (S), the Markov chain transitions to the central silent state. From here it may terminate by transitioning to the end state (E), or it may enter a match (+/+), insert (-/+) or delete (+/-) fragment. Each fragment has probability *δ*(*t*) of being an insert or delete fragment. Fragment lengths are geometric with continuation probability *ε*. After the end of a fragment, the Markov chain returns to the central silent state where it may begin a new fragment. The silent state that indicates fragment boundaries can be removed, resulting in transitions only between non-silent states. The model is a fragment based model because the direct transition probability from (+/+) to (+/+) without going through the silent state is *ε *and not 0. The pair-HMM represents an improper distribution because the probabilities of outgoing edges of the central silent state do not sum to 1.

This model can be expressed as a symmetrical pair-HMM (Figure 1), implying that alignments can be considered non-directed, since the probability does not change when ancestor and descendant sequences are interchanged. This contrasts with the TKF models that induce irreversible distributions on pairwise alignments. A major advantage of this symmetry is that it is clear how to construct alignment models on an unrooted tree and leads to greater simplicity in model implementation and, arguably, decreased computation time. The model described here diverges from our previous model in that match fragments no longer contain only a single letter, but instead follow the same length distribution as gap fragments. This is represented graphically in the pair-HMM by the addition of a loop with non-zero weight *ε *from the match state (+/+) to itself.

To facilitate dependence of pairwise alignment distribution *ν*_*t *_on *t*, we seek a natural relationship between *δ*(*t*) and *t*. We define *λ *as the indel rate per residue scaled in terms of substitution time and refer to the pairwise alignment distribution on branch *b *as ν(b)=νλtb
 MathType@MTEF@5@5@+=feaafiart1ev1aaatCvAUfKttLearuWrP9MDH5MBPbIqV92AaeXatLxBI9gBaebbnrfifHhDYfgasaacH8akY=wiFfYdH8Gipec8Eeeu0xXdbba9frFj0=OqFfea0dXdd9vqai=hGuQ8kuc9pgc9s8qqaq=dirpe0xb9q8qiLsFr0=vr0=vr0dc8meaabaqaciaacaGaaeqabaqabeGadaaakeaaiiGacqWF9oGBdaahaaWcbeqaaiabcIcaOiabdkgaIjabcMcaPaaakiabg2da9iab=17aUnaaBaaaleaacqWF7oaBcqWG0baDdaWgaaadbaGaemOyaigabeaaaSqabaaaaa@392B@. The parameter *ε *remains independent of time. In our previous model, we measure the occurrence probability of indels on a per-residue basis. In the fragment-based model, *δ'*(*t*) becomes the probability of a fragment being inserted or deleted. We wish to re-parameterize the fragment model in terms of a per-residue indel rate; the probability of an indel occurring between two residues is (1 - *ε*)*δ'*(*t*). However, if we attempt to set

(1−ε)δ′(t)=1−e−λtb,     (4)
 MathType@MTEF@5@5@+=feaafiart1ev1aaatCvAUfKttLearuWrP9MDH5MBPbIqV92AaeXatLxBI9gBaebbnrfifHhDYfgasaacH8akY=wiFfYdH8Gipec8Eeeu0xXdbba9frFj0=OqFfea0dXdd9vqai=hGuQ8kuc9pgc9s8qqaq=dirpe0xb9q8qiLsFr0=vr0=vr0dc8meaabaqaciaacaGaaeqabaqabeGadaaakeaacqGGOaakcqaIXaqmcqGHsisliiGacqWF1oqzcqGGPaqkcuWF0oazgaqbaiabcIcaOiabdsha0jabcMcaPiabg2da9iabigdaXiabgkHiTiabdwgaLnaaCaaaleqabaGaeyOeI0Iae83UdWMaemiDaq3aaSbaaWqaaiabdkgaIbqabaaaaOGaeiilaWIaaCzcaiaaxMaadaqadaqaaiabisda0aGaayjkaiaawMcaaaaa@454F@

then the probability *δ'*(*t*) can become greater than 1. We therefore move the factor of (1 - *ε*) into the time scale, such that

δ′(t)=1−e−λtb1−ε.     (5)
 MathType@MTEF@5@5@+=feaafiart1ev1aaatCvAUfKttLearuWrP9MDH5MBPbIqV92AaeXatLxBI9gBaebbnrfifHhDYfgasaacH8akY=wiFfYdH8Gipec8Eeeu0xXdbba9frFj0=OqFfea0dXdd9vqai=hGuQ8kuc9pgc9s8qqaq=dirpe0xb9q8qiLsFr0=vr0=vr0dc8meaabaqaciaacaGaaeqabaqabeGadaaakeaaiiGacuWF0oazgaqbaiabcIcaOiabdsha0jabcMcaPiabg2da9iabigdaXiabgkHiTiabdwgaLnaaCaaaleqabaGaeyOeI0YaaSaaaeaacqWF7oaBcqWG0baDdaWgaaadbaGaemOyaigabeaaaSqaaiabigdaXiabgkHiTiab=v7aLbaaaaGccqGGUaGlcaWLjaGaaCzcamaabmaabaGaeGynaudacaGLOaGaayzkaaaaaa@43BE@

We note that Equation (4) agrees with Equation (5) to first order in *λt*_*b *_and serves to connect fragment indel rates to per-residue indel rates. The product *λt*_*b *_is in general ≪ 1, so matching on higher order terms is unnecessary.

The distribution *ν*_*t *_naturally gives rise to two models. In the first model, denoted "fragments", we set *ν*^(*b*) ^= *ν*_*λ *_for all *b*, making the probability f an indel independent of branch length again. In the second model, denoted as "fragments+*T*", we set ν(b)=νλtb
 MathType@MTEF@5@5@+=feaafiart1ev1aaatCvAUfKttLearuWrP9MDH5MBPbIqV92AaeXatLxBI9gBaebbnrfifHhDYfgasaacH8akY=wiFfYdH8Gipec8Eeeu0xXdbba9frFj0=OqFfea0dXdd9vqai=hGuQ8kuc9pgc9s8qqaq=dirpe0xb9q8qiLsFr0=vr0=vr0dc8meaabaqaciaacaGaaeqabaqabeGadaaakeaaiiGacqWF9oGBdaahaaWcbeqaaiabcIcaOiabdkgaIjabcMcaPaaakiabg2da9iab=17aUnaaBaaaleaacqWF7oaBcqWG0baDdaWgaaadbaGaemOyaigabeaaaSqabaaaaa@392B@ making the probability of an indel roughly proportional to branch length *t*_*b*_.

We now show that the sequence length distribution induced by *ν*_*t *_is independent of *t*. The pairwise alignment distribution is a uniform distribution on the number of fragments, with each fragment being a match (+/+), insertion (-/+) or deletion (+/-) with probabilities 1 - 2*δ*(*t*), *δ*(*t*) and *δ*(*t*), respectively, and with exit measure (1 - *δ*(*t*)). This results in the following probability generating function for the length of either sequence in the pair-HMM:

f(s)=1−ε1−s+ε.     (6)
 MathType@MTEF@5@5@+=feaafiart1ev1aaatCvAUfKttLearuWrP9MDH5MBPbIqV92AaeXatLxBI9gBaebbnrfifHhDYfgasaacH8akY=wiFfYdH8Gipec8Eeeu0xXdbba9frFj0=OqFfea0dXdd9vqai=hGuQ8kuc9pgc9s8qqaq=dirpe0xb9q8qiLsFr0=vr0=vr0dc8meaabaqaciaacaGaaeqabaqabeGadaaakeaacqWGMbGzcqGGOaakcqWGZbWCcqGGPaqkcqGH9aqpdaWcaaqaaiabigdaXiabgkHiTGGaciab=v7aLbqaaiabigdaXiabgkHiTiabdohaZbaacqGHRaWkcqWF1oqzcqGGUaGlcaWLjaGaaCzcamaabmaabaGaeGOnaydacaGLOaGaayzkaaaaaa@403E@

Therefore, the length distribution is independent of *δ*(*t*), and is uniform except for an anomaly at length 0. This allows us to specify a different value of *δ*(*t*) in the pair-HMM on each branch of the tree without affecting *φ*. Defining *L*_1 _and *L*_2 _as the emitted sequence lengths from the pair-HMM, we note that *P*(*L*_1 _= *l*_1_) has finite measure and that the distribution *P*(*L*_2 _= *l*_2_|*L*_1 _= *l*_1_) on *L*_2 _is therefore proper. This implies that the posterior distribution of the joint model is proper because the distribution conditions on the observed leaf sequence lengths.

#### Sampling

**Figure 2:**
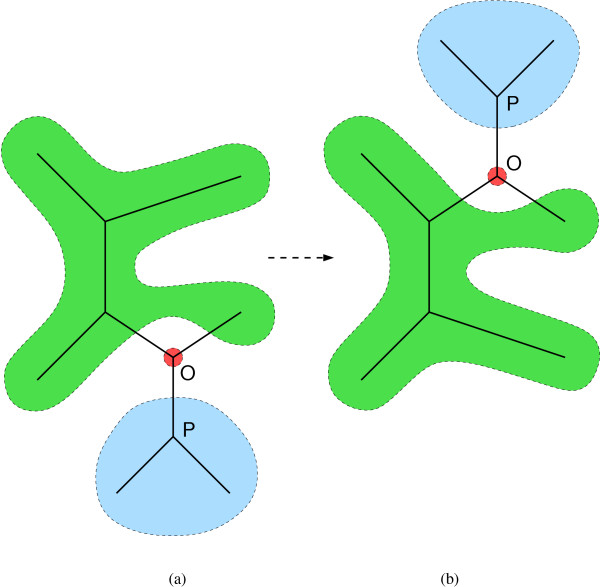
**The subtree-prune-and-regraft operator**. (a) First a subtree (blue) and its associated node O are detached from the rest of the tree (green). (b) The subtree is then regrafted along into a different branch through its node O. In both (a) and (b), three branches connect to node O. The phylogeny relating sequences at the pruned nodes (blue) and the phylogeny relating sequences at the remaining nodes (green) do not change. Therefore alignments within each of these sequence subsets can remain unchanged from (a) to (b).

We introduce a novel MCMC transition kernel that improves mixing between topologies and alignments. The new transition kernel uses the SPR operator (Figure 2) to propose new trees, but is extended to be alignment-aware. Our previous approach used only nearest-neighbor-interchange (NNI) operators to propose new trees [24]. This resulted in long convergence times and inefficient mixing when there were many taxa. The SPR operator improves on this situation by proposing non-local topology rearrangements that would require several NNI moves, and thus avoids several intermediates [27].

The extended SPR transition kernel updates the alignment **A **along with the topology *τ*. In our framework, it is necessary to alter **A **when *τ *is altered because **A **specifies the homology of internal sequences and this homology may be inconsistent with the proposed topology. This happens when some column of **A **contains a letter that would be deleted and reinserted given the new topology. After an SPR tree proposal, we note that the alignment of the subset of sequences corresponding to taxa in the pruned subtree (Figure 2, blue) must remain consistent because their phylogeny remains unchanged. Likewise, the alignment of the other sequences (Figure 2, green) must remain consistent because the phylogeny of that subset remains unchanged. However the alignment of the complete set of sequences may not be consistent.

Our solution to this problem involves collapsed Gibbs sampling [28] of **A **as follows. We define the collapsed point (*, *τ*, **T**, **Θ**, **Λ**)_*C *_as the set of points {(**A**, *τ*, **T**, **Θ**, **Λ**) : **A **∈ *C*} for some set *C*. The posterior probability of a collapsed point is then naturally defined as the posterior probability of the set. When proposing a new tree (*τ'*, **T***'*) via SPR from the current point (**A**, *τ*, **T**, **Θ**, **Λ**), we first use a Metropolis-Hasting (MH) transition kernel to choose between the collapsed points (*, *τ*, **T**, **Θ**, **Λ**)_*C *_and (*, *τ'*, **T***'*, **Θ**, **Λ**)_*C*_. This avoids the problem of **A **being inconsistent with *τ' *as long as *C *is large enough to contain alignments consistent with both *τ *and *τ'*. Then we sample a single point from the chosen collapsed point in proportion to its posterior probability. To satisfy detailed balance, the set *C *must be constructed so that it contains at least the current alignment **A**; full conditions under which this procedure satisfies detailed balance are described in the Appendix.

We now seek a set *C *that is large enough to contain alignments consistent with *τ' *and yet small enough for integration and sampling to be computationally feasible. Unfortunately, integration over the set of all alignments is not practical, even if we constrain the alignment of leaf sequences to be constant. Therefore, we fix parts of the alignment and collapse only the remaining portions. Allowing only the three branch alignments adjacent to node O (Figure 2) to vary will certainly allow an alignment consistent with *τ'*. This is therefore a loose constraint, which we call *C*_3_(**A**, *τ*, **O**). It requires an *O*(*L*^3^) dynamic programming algorithm for integration and resampling. To decrease the order of the dynamic programming algorithm to *O*(*L*^1^), we consider imposing the additional constraint, which we call *C*_1_(**A**, *τ*, **O**), that the alignments between the three nodes connected to **O **remain fixed. However, this constraint is too tight because it forces all sequences in the subtree (Figure 2, hatched) to keep the same alignment with the remaining sequences, and may not include any alignments consistent with *τ'*. As an alternative, we propose to fix the alignment between sequences in the pruned subtree and the alignment between sequences in the remainder, but allow the alignment between the two groups of sequences to vary. This constraint, which we call *C*_2_(**A**, *τ*, OP) results in an *O*(*L*^2^) algorithm that is significantly more computationally efficient than an *O*(*L*^3^) algorithm. Note that we have demonstrated above that the alignment within the two subgroups of sequences remains consistent under an SPR proposal. Thus, the constraint set *C*_2_(**A**, *τ*, OP) contains an alignment that is consistent with *τ' *as well as *τ*, making *C*_2_(**A**, *τ*, OP) a useful constraint set for collapsed sampling.

#### Triplet Models

Triplet models coalesce three adjacent nucleotide letters into a single triplet letter. The size of the triplet alphabet is therefore approximately the cube of the size of the singlet alphabet. The larger alphabet size allows a more complex substitution model such as the codon model of Goldman and Yang [[25], M0]. Triplet substitution models can prohibit stop codons, can make use of codon frequencies instead of nucleotide frequencies and can differentiate between synonymous and non-synonymous substitutions. Triplet alphabets affect the alignment model as well as the substitution model by forcing indels lengths to be multiples of 3 singlet letters and by forcing indels to start and end between triplets. While the former is biologically realistic, the latter may not be.

We describe a method of comparing triplet with singlet models to assess how forcing indels to begin and end between codons affects model fit. To accomplish this, we first remove the substitution benefits of the M0 model listed above to focus solely on the effects of the triplet alignment process. We construct a triplet substitution model that generates the same likelihood as a singlet substitution model given the same alignment. Traditionally, both models are reversible and have a rate matrix **Q **= {*Q*_*xy*_} that is constructed from the equilibrium letter frequencies *π *and a symmetric exchangeability matrix **S **= {*S*_*xy*_} in the following way:

Qxy=Sxyπyfπx1−f.     (7)
 MathType@MTEF@5@5@+=feaafiart1ev1aaatCvAUfKttLearuWrP9MDH5MBPbIqV92AaeXatLxBI9gBaebbnrfifHhDYfgasaacH8akY=wiFfYdH8Gipec8Eeeu0xXdbba9frFj0=OqFfea0dXdd9vqai=hGuQ8kuc9pgc9s8qqaq=dirpe0xb9q8qiLsFr0=vr0=vr0dc8meaabaqaciaacaGaaeqabaqabeGadaaakeaacqWGrbqudaWgaaWcbaGaemiEaGNaemyEaKhabeaakiabg2da9iabdofatnaaBaaaleaacqWG4baEcqWG5bqEaeqaaOWaaSaaaeaaiiGacqWFapaCdaqhaaWcbaGaemyEaKhabaGaemOzaygaaaGcbaGae8hWda3aa0baaSqaaiabdIha4bqaaiabigdaXiabgkHiTiabdAgaMbaaaaGccqGGUaGlcaWLjaGaaCzcamaabmaabaGaeG4naCdacaGLOaGaayzkaaaaaa@4682@

Fraction *f *can vary from 0 to 1 but traditionally *f *is fixed to 1. The fraction specifies the relative importance of unequal conservation (*f *= 0) and unequal replacement (*f *= 1) in creating the equilibrium frequency distribution [29].

Given a singlet nucleotide model with exchangeability matrix **S**^(*s*)^, we build a triplet model with exchangeability matrix **S**^(*t*) ^in the following fashion. Each allowable substitution from triplet *α *to triplet *β *involves only one nucleotide substitution from nucleotide *i *to nucleotide *j*. Therefore, we set Sαβ(t)=Sij(s)
 MathType@MTEF@5@5@+=feaafiart1ev1aaatCvAUfKttLearuWrP9MDH5MBPbIqV92AaeXatLxBI9gBaebbnrfifHhDYfgasaacH8akY=wiFfYdH8Gipec8Eeeu0xXdbba9frFj0=OqFfea0dXdd9vqai=hGuQ8kuc9pgc9s8qqaq=dirpe0xb9q8qiLsFr0=vr0=vr0dc8meaabaqaciaacaGaaeqabaqabeGadaaakeaacqWGtbWudaqhaaWcbaacciGae8xSdeMae8NSdigabaGaeiikaGIaemiDaqNaeiykaKcaaOGaeyypa0Jaem4uam1aa0baaSqaaiabdMgaPjabdQgaQbqaaiabcIcaOiabdohaZjabcMcaPaaaaaa@3CB2@ in this case, and Sαβ(t)
 MathType@MTEF@5@5@+=feaafiart1ev1aaatCvAUfKttLearuWrP9MDH5MBPbIqV92AaeXatLxBI9gBaebbnrfifHhDYfgasaacH8akY=wiFfYdH8Gipec8Eeeu0xXdbba9frFj0=OqFfea0dXdd9vqai=hGuQ8kuc9pgc9s8qqaq=dirpe0xb9q8qiLsFr0=vr0=vr0dc8meaabaqaciaacaGaaeqabaqabeGadaaakeaacqWGtbWudaqhaaWcbaacciGae8xSdeMae8NSdigabaGaeiikaGIaemiDaqNaeiykaKcaaaaa@346D@ = 0 for all other entries. If the singlet model is, for example, the model of Hasegawa et al. [[30], HKY], we term the resulting triplet model as HKY × 3. We also set the triplet frequencies πα(t)
 MathType@MTEF@5@5@+=feaafiart1ev1aaatCvAUfKttLearuWrP9MDH5MBPbIqV92AaeXatLxBI9gBaebbnrfifHhDYfgasaacH8akY=wiFfYdH8Gipec8Eeeu0xXdbba9frFj0=OqFfea0dXdd9vqai=hGuQ8kuc9pgc9s8qqaq=dirpe0xb9q8qiLsFr0=vr0=vr0dc8meaabaqaciaacaGaaeqabaqabeGadaaakeaaiiGacqWFapaCdaqhaaWcbaGae8xSdegabaGaeiikaGIaemiDaqNaeiykaKcaaaaa@335A@ for each triplet *α *composed of nucleotides *i*, *j *and *k *to the product πi(s)πj(s)πk(s)
 MathType@MTEF@5@5@+=feaafiart1ev1aaatCvAUfKttLearuWrP9MDH5MBPbIqV92AaeXatLxBI9gBaebbnrfifHhDYfgasaacH8akY=wiFfYdH8Gipec8Eeeu0xXdbba9frFj0=OqFfea0dXdd9vqai=hGuQ8kuc9pgc9s8qqaq=dirpe0xb9q8qiLsFr0=vr0=vr0dc8meaabaqaciaacaGaaeqabaqabeGadaaakeaaiiGacqWFapaCdaqhaaWcbaGaemyAaKgabaGaeiikaGIaem4CamNaeiykaKcaaOGae8hWda3aa0baaSqaaiabdQgaQbqaaiabcIcaOiabdohaZjabcMcaPaaakiab=b8aWnaaDaaaleaacqWGRbWAaeaacqGGOaakcqWGZbWCcqGGPaqkaaaaaa@3FF5@ of the individual nucleotide frequencies.

Although this construction might be expected to yield a triplet substitution model that is identical to the singlet substitution model, this is not the case if *f *= 1. For an allowable substitution *ijk *→ *ijl*, we note that the rate Qijk,ijl(t)
 MathType@MTEF@5@5@+=feaafiart1ev1aaatCvAUfKttLearuWrP9MDH5MBPbIqV92AaeXatLxBI9gBaebbnrfifHhDYfgasaacH8akY=wiFfYdH8Gipec8Eeeu0xXdbba9frFj0=OqFfea0dXdd9vqai=hGuQ8kuc9pgc9s8qqaq=dirpe0xb9q8qiLsFr0=vr0=vr0dc8meaabaqaciaacaGaaeqabaqabeGadaaakeaacqWGrbqudaqhaaWcbaGaemyAaKMaemOAaOMaem4AaSMaeiilaWIaemyAaKMaemOAaOMaemiBaWgabaGaeiikaGIaemiDaqNaeiykaKcaaaaa@3A37@ according to the triplet model does not match the rate Qkl(s)=Skl(s)πl(s)
 MathType@MTEF@5@5@+=feaafiart1ev1aaatCvAUfKttLearuWrP9MDH5MBPbIqV92AaeXatLxBI9gBaebbnrfifHhDYfgasaacH8akY=wiFfYdH8Gipec8Eeeu0xXdbba9frFj0=OqFfea0dXdd9vqai=hGuQ8kuc9pgc9s8qqaq=dirpe0xb9q8qiLsFr0=vr0=vr0dc8meaabaqaciaacaGaaeqabaqabeGadaaakeaacqWGrbqudaqhaaWcbaGaem4AaSMaemiBaWgabaGaeiikaGIaem4CamNaeiykaKcaaOGaeyypa0Jaem4uam1aa0baaSqaaiabdUgaRjabdYgaSbqaaiabcIcaOiabdohaZjabcMcaPaaaiiGakiab=b8aWnaaDaaaleaacqWGSbaBaeaacqGGOaakcqWGZbWCcqGGPaqkaaaaaa@42AF@ according to the singlet model. Specifically,

Qijk,ijl(t)=Sijk,ijl(t)πijl(t)=Skl(s)πi(s)πj(s)πl(s)≠Qkl(s).     (8)
 MathType@MTEF@5@5@+=feaafiart1ev1aaatCvAUfKttLearuWrP9MDH5MBPbIqV92AaeXatLxBI9gBaebbnrfifHhDYfgasaacH8akY=wiFfYdH8Gipec8Eeeu0xXdbba9frFj0=OqFfea0dXdd9vqai=hGuQ8kuc9pgc9s8qqaq=dirpe0xb9q8qiLsFr0=vr0=vr0dc8meaabaqaciaacaGaaeqabaqabeGadaaakeaafaqaaeWadaaabaGaemyuae1aa0baaSqaaiabdMgaPjabdQgaQjabdUgaRjabcYcaSiabdMgaPjabdQgaQjabdYgaSbqaaiabcIcaOiabdsha0jabcMcaPaaaaOqaaiabg2da9aqaaiabdofatnaaDaaaleaacqWGPbqAcqWGQbGAcqWGRbWAcqGGSaalcqWGPbqAcqWGQbGAcqWGSbaBaeaacqGGOaakcqWG0baDcqGGPaqkaaacciGccqWFapaCdaqhaaWcbaGaemyAaKMaemOAaOMaemiBaWgabaGaeiikaGIaemiDaqNaeiykaKcaaaGcbaaabaGaeyypa0dabaGaem4uam1aa0baaSqaaiabdUgaRjabdYgaSbqaaiabcIcaOiabdohaZjabcMcaPaaakiab=b8aWnaaDaaaleaacqWGPbqAaeaacqGGOaakcqWGZbWCcqGGPaqkaaGccqWFapaCdaqhaaWcbaGaemOAaOgabaGaeiikaGIaem4CamNaeiykaKcaaOGae8hWda3aa0baaSqaaiabdYgaSbqaaiabcIcaOiabdohaZjabcMcaPaaaaOqaaaqaaiabgcMi5cqaaiabdgfarnaaDaaaleaacqWGRbWAcqWGSbaBaeaacqGGOaakcqWGZbWCcqGGPaqkaaGccqGGUaGlaaGaaCzcaiaaxMaadaqadaqaaiabiIda4aGaayjkaiaawMcaaaaa@7B7C@

The rates do not match because the rate of change from *k *→ *l *in the triplet model depends on the frequencies of the other nucleotides in the triplet. Since this is not true in the singlet model, the likelihoods under each model cannot match unless all the nucleotide frequencies are equal.

However, removing the constraint that *f *= 1, it becomes possible for the two models to coalesce because the rate of change Qijk,ijl(t)
 MathType@MTEF@5@5@+=feaafiart1ev1aaatCvAUfKttLearuWrP9MDH5MBPbIqV92AaeXatLxBI9gBaebbnrfifHhDYfgasaacH8akY=wiFfYdH8Gipec8Eeeu0xXdbba9frFj0=OqFfea0dXdd9vqai=hGuQ8kuc9pgc9s8qqaq=dirpe0xb9q8qiLsFr0=vr0=vr0dc8meaabaqaciaacaGaaeqabaqabeGadaaakeaacqWGrbqudaqhaaWcbaGaemyAaKMaemOAaOMaem4AaSMaeiilaWIaemyAaKMaemOAaOMaemiBaWgabaGaeiikaGIaemiDaqNaeiykaKcaaaaa@3A37@ can be independent of the frequencies of *i *and *j*. Setting *f *= 12
 MathType@MTEF@5@5@+=feaafiart1ev1aaatCvAUfKttLearuWrP9MDH5MBPbIqV92AaeXatLxBI9gBaebbnrfifHhDYfgasaacH8akY=wiFfYdH8Gipec8Eeeu0xXdbba9frFj0=OqFfea0dXdd9vqai=hGuQ8kuc9pgc9s8qqaq=dirpe0xb9q8qiLsFr0=vr0=vr0dc8meaabaqaciaacaGaaeqabaqabeGadaaakeaadaWcaaqaaiabigdaXaqaaiabikdaYaaaaaa@2E9E@, the frequencies of neighboring nucleotides no longer affect the rate of change from *k *→ *l*, as

Qijk,ijl(t)=Sijk,ijl(t)πijl(t)πijk(t)=Skl(s)πi(s)πj(s)πl(s)πi(s)πj(s)πk(s)=Skl(s)πl(s)πk(s).     (9)
 MathType@MTEF@5@5@+=feaafiart1ev1aaatCvAUfKttLearuWrP9MDH5MBPbIqV92AaeXatLxBI9gBaebbnrfifHhDYfgasaacH8akY=wiFfYdH8Gipec8Eeeu0xXdbba9frFj0=OqFfea0dXdd9vqai=hGuQ8kuc9pgc9s8qqaq=dirpe0xb9q8qiLsFr0=vr0=vr0dc8meaabaqaciaacaGaaeqabaqabeGadaaakeaafaqaaeGadaaabaGaemyuae1aa0baaSqaaiabdMgaPjabdQgaQjabdUgaRjabcYcaSiabdMgaPjabdQgaQjabdYgaSbqaaiabcIcaOiabdsha0jabcMcaPaaaaOqaaiabg2da9aqaaiabdofatnaaDaaaleaacqWGPbqAcqWGQbGAcqWGRbWAcqGGSaalcqWGPbqAcqWGQbGAcqWGSbaBaeaacqGGOaakcqWG0baDcqGGPaqkaaGcdaGcaaqaamaalaaabaacciGae8hWda3aa0baaSqaaiabdMgaPjabdQgaQjabdYgaSbqaaiabcIcaOiabdsha0jabcMcaPaaaaOqaaiab=b8aWnaaDaaaleaacqWGPbqAcqWGQbGAcqWGRbWAaeaacqGGOaakcqWG0baDcqGGPaqkaaaaaaqabaaakeaaaeaacqGH9aqpaeaacqWGtbWudaqhaaWcbaGaem4AaSMaemiBaWgabaGaeiikaGIaem4CamNaeiykaKcaaOWaaOaaaeaadaWcaaqaaiab=b8aWnaaDaaaleaacqWGPbqAaeaacqGGOaakcqWGZbWCcqGGPaqkaaGccqWFapaCdaqhaaWcbaGaemOAaOgabaGaeiikaGIaem4CamNaeiykaKcaaOGae8hWda3aa0baaSqaaiabdYgaSbqaaiabcIcaOiabdohaZjabcMcaPaaaaOqaaiab=b8aWnaaDaaaleaacqWGPbqAaeaacqGGOaakcqWGZbWCcqGGPaqkaaGccqWFapaCdaqhaaWcbaGaemOAaOgabaGaeiikaGIaem4CamNaeiykaKcaaOGae8hWda3aa0baaSqaaiabdUgaRbqaaiabcIcaOiabdohaZjabcMcaPaaaaaaabeaakiabg2da9iabdofatnaaDaaaleaacqWGRbWAcqWGSbaBaeaacqGGOaakcqWGZbWCcqGGPaqkaaGcdaGcaaqaamaalaaabaGae8hWda3aa0baaSqaaiabdYgaSbqaaiabcIcaOiabdohaZjabcMcaPaaaaOqaaiab=b8aWnaaDaaaleaacqWGRbWAaeaacqGGOaakcqWGZbWCcqGGPaqkaaaaaaqabaGccqGGUaGlaaGaaCzcaiaaxMaadaqadaqaaiabiMda5aGaayjkaiaawMcaaaaa@A46D@

We note that for branch lengths to agree between the singlet and triplet models, **Q**^(*t*) ^must be scaled so that ∑α≠βπα(t)Qαβ(t)
 MathType@MTEF@5@5@+=feaafiart1ev1aaatCvAUfKttLearuWrP9MDH5MBPbIqV92AaeXatLxBI9gBaebbnrfifHhDYfgasaacH8akY=wiFfYdH8Gipec8Eeeu0xXdbba9frFj0=OqFfea0dXdd9vqai=hGuQ8kuc9pgc9s8qqaq=dirpe0xb9q8qiLsFr0=vr0=vr0dc8meaabaqaciaacaGaaeqabaqabeGadaaakeaadaaeqaqaaGGaciab=b8aWnaaDaaaleaacqWFXoqyaeaacqGGOaakcqWG0baDcqGGPaqkaaGccqWGrbqudaqhaaWcbaGae8xSdeMae8NSdigabaGaeiikaGIaemiDaqNaeiykaKcaaaqaaiab=f7aHjabgcMi5kab=j7aIbqab0GaeyyeIuoaaaa@41EA@ instead of the usual 1, because **Q**^(*t*) ^measures changes of each of the three sites in the triplet. We use HKY as the singlet model in our comparison because the HKY × 3 model is identical to the M0 codon model with *ω *= 1, stop codons included, and independent nucleotide frequencies.

### Examples

We analyze two data examples to demonstrate the advantages of joint Bayesian estimation. While both data sets come from related genes, they differ in their sequence lengths, number of taxa, and sequence characteristics. We select these datasets for their relative sparseness of phylogenetic information, typical of rapidly evolving pathogens. Thus, although the joint model makes full use of both indels and substitutions shared by descent, we do not expect to recover fully resolved trees. Rather, we note substantial improvement over traditional, sequential methods.

#### Example 1: SIV

We first examine a data set drawn from SIV, a non-human primate lentivirus. Lentiviruses contain a single-stranded RNA genome that reverse transcribes into DNA by upon infection. The DNA then inserts into the host genome before expression. Reverse transcriptase is extremely error-prone, giving lentiviruses high mutation rates. The data set consists of 9 partial *env *sequences sampled from within a single macaque initially infected by injection with strain SIVmac251 [31]. Cheynier et al (2001) have previously presented an alignment of these sequences as a typical example of phylogenetically informative indels in SIV [11].

The *env *gene encodes glycoprotein gp160, which is split after translation to form the smaller glycoproteins gp120 and gp41. Because gp120 and gp41 are displayed on the surface of mature virions, exposed to the host immune system, *env *tends to mutate more quickly than other SIV genes through positive selection. From the data set, we remove a phylogenetically uninformative duplication in a single sequence because our model assumes insertions of random sequence but not duplications. All sequences then range in length from 57 to 69 nucleotides with an alignment length of 69 nucleotides independent of the method used to compute the fixed alignment. The data set contains 10 variable sites and 6 informative sites under the Clustal W alignment, 12 variable and 7 informative sites under the Muscle alignment, and 11 variable and 6 informative sites under the MAP estimate from the joint model (Table 1, – Indel contribution).

**Table 1:** Phylogenetic resolution of various models in SIV.

Model	**Â**	# sites		#/6 with PP			Estimates		
		var.	inf.	> 0.90	> 0.95	> 0.99	*κ*	ln *λ*	ln *ε*
- Indel	Clustal W	10	6	1	1	1	2.3(1.6,4.9)	-	-
	Muscle	12	7	4	3	0	2.2(1.5,4.3)	-	-
	MAP	11	6	3	1	1	2.4(1.6,5.2)	-	-

+ Indel	Clustal W	10	6	3+1	3+1	2+1	2.3(1.6,4.7)	-2.7(-4.5,-1.4)	-0.61(-0.93,-0.37)
	Muscle	12	7	3	3	2	2.3(1.6,4.4)	-3.5(-5.0,-2.1)	-0.92(-1.5,-0.55)
	MAP	11	6	5	4	3	2.4(1.6,5.1)	-3.4(-5.0,-1.9)	-0.71(-1.1,-0.43)

Joint	-	-	-	4	3	3	2.4(1.6,5.3)	-3.4(-5.0,-1.9)	-0.71(-1.1,-0.43)

Results are presented for the traditional sequential model (- Indel), the joint model with a fixed alignment (+ Indel), and the full joint model. The choice of a fixed alignment estimate is indicated in column **Â **if applicable. The number of variable and informative sites is indicated under "#sites" and varies depending on the choice of alignment. The number of supported internal branches out of a possible 6 is reported at three levels of posterior probability (PP). The designation "+1" indicates that 1 of the supported branches conflicts with a branch supported under the joint model. Estimates of continuous parameters *κ*, ln *λ*, and ln *ε *are presented as a posterior median followed by a 95% Bayesian credible interval.

For a prior on ln *κ*, we assume a Double-Exponential distribution with median ln 2 and standard deviation 14
 MathType@MTEF@5@5@+=feaafiart1ev1aaatCvAUfKttLearuWrP9MDH5MBPbIqV92AaeXatLxBI9gBaebbnrfifHhDYfgasaacH8akY=wiFfYdH8Gipec8Eeeu0xXdbba9frFj0=OqFfea0dXdd9vqai=hGuQ8kuc9pgc9s8qqaq=dirpe0xb9q8qiLsFr0=vr0=vr0dc8meaabaqaciaacaGaaeqabaqabeGadaaakeaadaWcaaqaaiabigdaXaqaaiabisda0aaaaaa@2EA2@. On ln *λ*, we assume a Double-Exponential distribution with median -5 and standard deviation 12
 MathType@MTEF@5@5@+=feaafiart1ev1aaatCvAUfKttLearuWrP9MDH5MBPbIqV92AaeXatLxBI9gBaebbnrfifHhDYfgasaacH8akY=wiFfYdH8Gipec8Eeeu0xXdbba9frFj0=OqFfea0dXdd9vqai=hGuQ8kuc9pgc9s8qqaq=dirpe0xb9q8qiLsFr0=vr0=vr0dc8meaabaqaciaacaGaaeqabaqabeGadaaakeaadaWcaaqaaiabigdaXaqaaiabikdaYaaaaaa@2E9E@. For *ε *we assume an Exponential distribution with mean 5 on the expected indel length. We assume a Uniform distribution over the topology *τ*. On the branch lengths we assume an Exponential distribution with mean *μ*, and on *μ *we assume an Exponential distribution with mean 0.04. Continuous parameter estimates under the joint model are as follows: *κ *has median 2.4 with a 95% Bayesian credible interval of (1.64,5.32). The median of ln *λ *is -3.4 and its 95% BCI is (-4.99, -1.85). The median of ln *ε *is -0.71 with a 95% BCI of (-1.12, -0.428). The mean branch length *μ*, has posterior median 0.0178 with a 95% BCI of (0.00854,0.0368).

**Figure 3:**
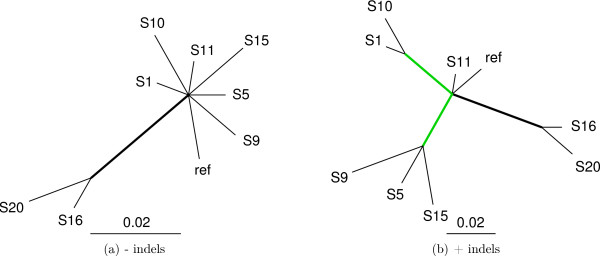
**Indel information improves resolution of the SIV phylogeny**. (a) At posterior probability > 0.99 the traditional sequential model supports only one branch, (b) When indel information is included, the number of supported branches rises to 3. The two green branches are supported only when indel information is used.

To assess the usefulness of indel information and the importance of alignment ambiguity in phylogenetic inference, we compare the posterior topology distributions for the traditional sequential model, the joint model restricted to a fixed alignment, and the full joint model. We note that the joint model increases the number of resolved internal branches by 3, 2, and 2 at posterior probability (PP) > 0.9, > 0.95, and > 0.99, respectively, over the traditional model using the Clustal W alignment. The joint model supports 4, 3, and 3 branches at these levels of posterior probability and we depict the tree with branches supported at PP > 0.99 in Figure 3. This increase in resolution is sensitive to the alignment estimation method. For example, the resolution increase changes to 0, 0, and 2 under the Muscle alignment, and 1, 2, 2 under the joint MAP alignment. Thus, even accounting for alignment uncertainty, we achieve an increase in the phylogenetic resolution. At high posterior probabilities indels become relatively more important because they are rarer than substitutions.

**Figure 4:**
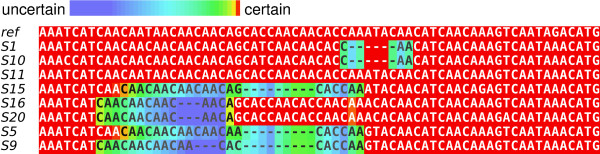
**SIV Alignment uncertainty plot**. We annotate the joint maximum a posteriori alignment estimate to indicate the approximate probability that each letter aligns to the root taxon in its column [24]. The 8 gaps in the alignment are a result of only 4 indel events under the joint model, whereas the Clustal W alignment requires at least 5 indel events. Colors other than red indicates that letters or gaps may shift to adjacent positions. The high frequency of the CAA triplet is partially responsible for the level of alignment uncertainty.

We note that alignment ambiguity is significant in this data set. First, estimates under the traditional or restricted models are sensitive to the alignment method used (Table 1). Second, fixing the alignment under the joint model yields an increase in the number of supported branches if the alignment is fixed to the Clustal W estimate or the joint MAP estimate, but a decrease if the Muscle estimate is used. Furthermore, the increased support when the Clustal W alignment is used includes a branch that conflicts with the joint MAP model, and the conflicting branch is present in the guide tree. Thus ignoring alignment ambiguity can lead to exaggerated support for branches and bias towards the guide tree, especially when indel information is used. Figure 4 displays a "gold" plot [24] to summarize the posterior alignment distribution of **A **under the full joint model. We observe a high level of alignment uncertainty. This is borne out by the observation of only 4 unique indels under the full joint model, while the Clustal W alignment contains 5 indels. This difference is reflected in the lower estimate of *λ *under the full joint model and in the restricted models not using the Clustal W alignment (Table 1).

#### Example 2: HIV-1

Our second data set consists of comparatively longer sequences from HIV-1, a lentivirus closely related to SIV. We consider a collection of 27 partial *env *gene sequences sampled serially at three time points from patient 1 reported by Shankarappa et al [4]. Each sequence name consists of a unique identifying number prefixed by the number of weeks after infection that the sequence was sampled. For the sake of brevity we drop the prefix and use only the unique identifier, except where explicity noted. The sequences span HIV genome reference sites 7023–7637 and include about 280 nucleotides of gp120, covering the V3 loop, followed by 330 nucleotides of gp41. Sequence lengths vary from 603 to 612 nucleotides. The number of homologous sites depends on the method used to compute the fixed alignment. Using the alignment from the MAP point or the Muscle alignment, this data set contains 621 columns, of which 76 are variable and 25 are informative. The Clustal W estimate generates an alignment of 618 columns, of which 77 are variable and 25 are informative.

##### Sequence Characteristics

We first analyze these data using the M0 codon model [25] to assess the importance of selection in this region (Table 2). We use the same prior distributions on *λ*, *ε*, *κ*, *τ*, and **T **as in Example 1. We additionally place a Double-Exponential distribution on ln *ω *with median 0 and standard deviation 0.1. In addition to the standard M0 model in which *f *is fixed to 1, we consider the case in which *f *is a random variable with a Uniform prior distribution. The posterior distribution of *ω *has median 0.996 and a 95% BCI of (0.834,1.20). This changes little when *f *is free. The estimated interval is quite close to the prior 95% BCI of (0.84,1.16) so we conclude that these data possess little information about *ω*. Allowing *ω *to vary does not yield much benefit, and we henceforth consider only *ω *= 1.

**Table 2:** Comparison of alignment and substitution models.

Model	ln P(**Y**)	*κ*	ln *λ*	ln *ε*	*ω*	*f*	(10,12,18)
HKY	-1555.7	7.2(4.6,11.7)	-3.3(-4.1,-2.7)	-1.0(-1.3,-0.78)	-	0.5	0.96(25)
HKY × 3	-1579.8	7.5(4.8,12.2)	-2.3(-3.1,-1.6)	-2.7(-3.7,-1.9)	-	0.5	0.75(3.1)
M0	-1542.7	7.2(4.6,11.8)	-2.2(-3.0,-1.6)	-2.7(-3.7,-1.9)	1.0(0.86,1.2)	0.46(0.26,0.65)	0.92(12)

We compare the HKY singlet model, the HKY × 3 triplet model, and the M0 codon model, that forbids stop codons. For the first two models we fix independent nucleotide frequencies but for the M0 model we allow codon frequencies to vary. Continuous parameter estimates are presented as a posterior median followed by a 95% Bayesian credible interval if free, and a single value if fixed. The HKY model has a higher marginal probability than the HKY × 3 triplet model, indicating that not all indels start and end between codons. Removing stop codons and allowing codon frequencies to vary freely increases the marginal likelihood of the M0 model substantially. Despite these increases in marginal likelihood, the M0 model does not support the clade (10,12,18) as well as the singlet model.

We also note that fixing *f *= 12
 MathType@MTEF@5@5@+=feaafiart1ev1aaatCvAUfKttLearuWrP9MDH5MBPbIqV92AaeXatLxBI9gBaebbnrfifHhDYfgasaacH8akY=wiFfYdH8Gipec8Eeeu0xXdbba9frFj0=OqFfea0dXdd9vqai=hGuQ8kuc9pgc9s8qqaq=dirpe0xb9q8qiLsFr0=vr0=vr0dc8meaabaqaciaacaGaaeqabaqabeGadaaakeaadaWcaaqaaiabigdaXaqaaiabikdaYaaaaaa@2E9E@ instead of the traditional value of 1 produces a decrease in marginal likelihood of 2 log units for the HKY model and a substantial increase of 5 log units for the HKY × 3 model (Table 2). When *f *varies under the M0 model, the resulting model is supported over the *f *= 1 model by 7 log units of marginal likelihood. In addition, the posterior median of *f *is 0.43, close to the value of 12
 MathType@MTEF@5@5@+=feaafiart1ev1aaatCvAUfKttLearuWrP9MDH5MBPbIqV92AaeXatLxBI9gBaebbnrfifHhDYfgasaacH8akY=wiFfYdH8Gipec8Eeeu0xXdbba9frFj0=OqFfea0dXdd9vqai=hGuQ8kuc9pgc9s8qqaq=dirpe0xb9q8qiLsFr0=vr0=vr0dc8meaabaqaciaacaGaaeqabaqabeGadaaakeaadaWcaaqaaiabigdaXaqaaiabikdaYaaaaaa@2E9E@ that is used to compare the HKY and HKY × 3 models. We therefore assume *f *= 0.5 for the remainder of our analyses.

Under the HKY model we find that *κ *has a posterior median of 7.2 with a 95% BCI of (4.6,11.7). The posterior median of ln *λ *is -3.3 with a 95% BCI of (-4.1, -2.7) and ln *ε *has a median of -1.0 and a 95% BCI of (-1.3, -0.78). This estimate of *ε *corresponds to a mean indel length of 1.58 nucleotides. The posterior median of *μ*, is 0.0036 with a 95% BCI of (0.00257,0.00508).

##### Singlet versus Triplet models

**Figure 5:**
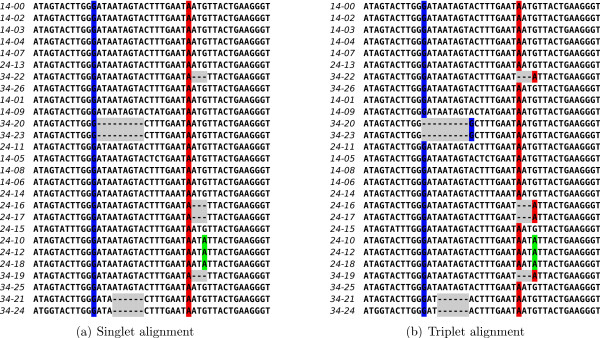
**Triplet alignments may shift indels and cause misaligned residues**. Triplet alignments may shift indels and cause misaligned residues. (a) Maximum a posteriori (MAP) alignment estimate under the singlet HKY model. (b) MAP alignment estimate under the triplet HKY × 3 model. In the triplet alignment, two G residues (blue) and four A residues (red) are forced into a different column to avoid breaking the alignment-wide reading frame. The displaced A residues join A residues from strains 10, 12, and 18 (green) which were previously the only A residues in that column. Under both models, the MAP alignment estimates display 8 gaps. The alignment of internal sequences (not shown) indicates that these gaps arose from 5 indel events on branches partitioning clades (20,23), (21,24), (16,17), (19), and (22). Thus, the gaps in sequences 19 and 22 arose independently of the gap in (16,17) even though they have the same length and position. Prefixes on sequence names indicate elapsed time in weeks between the initial infection and when the sequences were obtained.

To examine the model appropriateness of forcing indels to begin and end between codons, we compared the marginal likelihoods and posterior tree lengths for the HKY singlet and HKY × 3 triplet models. Under both models, we fixed *f *= 12
 MathType@MTEF@5@5@+=feaafiart1ev1aaatCvAUfKttLearuWrP9MDH5MBPbIqV92AaeXatLxBI9gBaebbnrfifHhDYfgasaacH8akY=wiFfYdH8Gipec8Eeeu0xXdbba9frFj0=OqFfea0dXdd9vqai=hGuQ8kuc9pgc9s8qqaq=dirpe0xb9q8qiLsFr0=vr0=vr0dc8meaabaqaciaacaGaaeqabaqabeGadaaakeaadaWcaaqaaiabigdaXaqaaiabikdaYaaaaaa@2E9E@ for equivalence and set independent nucleotide frequencies to their empirical estimates. The log marginal likelihood is -1555.7 ± 0.3 for the singlet model and -1579.8 ± 0.3 for the triplet model (Table 2). To examine the substantial decrease of 24.1 log units between models, we calculate the posterior distribution of parsimony tree lengths under both models. The posterior median tree length is 104 substitutions with a 95% BCI of (103,106) for the singlet model and increases to 109 substitutions with a 95% BCI of (108,110) for the triplet model. To verify that this increase results from forcing indels out of phase, we first calculate the posterior distribution of the number of indels under the singlet model. The posterior mean number of indels is 11.0 and the BCI is (11,11). The posterior mean number of indels beginning 0, 1, or 2 nucleotides from the beginning of a codon is 2.6, 5.8, and 2.6 respectively. The 95% BCI for the number of indels beginning inside a codon is (6,10). Inspecting the alignment estimate from the MAP point using a "gold" plot demonstrates alignment uncertainty (data not shown). In the MAP alignment we observe 11 indel events. Only 5 of the indels are consistently present with unambiguous phase, and none of these indels can be placed between codons. Interestingly, one indel of 3 nucleotides occurs independently in clades (16,17), 19, and 22 according to the MAP estimate (Figure 5). We note that augmented alignments such as those used in our model distinguish between indels shared by state and indel shared by descent through the inclusion of Felsenstein wildcard sequences at internal nodes of *τ*.

Use of the triplet model instead of the singlet model has a discernible effect on phylogeny estimation. The posterior odds in favor of the clade (10,12,18) decrease by a factor of 8.0 from 24.6 to 3.1 (Table 2). We note that in one column of the singlet MAP alignment estimate, only variants 10, 12 and 18 have an A residue, while other taxa have either a G residue or a gap (Figure 5a). However, the triplet model shifts these gaps out of the column to avoid breaking a codon. Taxa that contain a gap in this column under the singlet alignment contain A residues according to the triplet MAP alignment, decreasing the support for (10,12,18) clade (Figure 5b). Thus, comparing marginal likelihoods for model selection between the singlet and triplet models may not provide the whole picture.

Triplet models have discernible effects on estimates of the indel parameters *λ *and *ε*, but little effect on the substitution parameters *μ *and *κ*. For example, under the HKY × 3 model the posterior median of *λ *is *e*^-2.3^, about 3 times higher than the posterior median of *e*^-3.3 ^under the HKY model. We note that under the HKY × 3 model *λ *is the indel rate per triplet, whereas under the singlet model *λ *is the indel rate per nucleotide. This factor of 3 difference is to be expected since the number of indels does not change between the two models, but the number of triplets is 3 times smaller than the number of nucleotides. The HKY × 3 model also results in a posterior median estimate of -2.6 for ln *ε *that is significantly smaller than the HKY estimate of -1.0. However, accounting for the fact that one triplet contains three nucleotides, the HKY × 3 model predicts a mean indel length of 1.1 triplets and 3.2 nucleotides, but the HKY model predicts a mean indel length of 1.6 nucleotides. This may be because a geometric distribution on the number of nucleotides in a gap does not fit the data as well as a geometric distribution on the number of triplets in a gap. This is especially true in data sets such as the present one in which the number of triplets tends to be small. It may also be because the indel model used is fragment-based. Finally, we note that estimates of 7.5 for *κ *in the HKY × 3 model are quite similar to estimates of about 7.2 under the HKY model.

##### Increased support due to indel information

To assess how much indel information improves the resolution of the HIV phylogeny, we generate posterior samples under both the traditional, sequential model and under the full joint model. The traditional model supports 8, 7 and 4 internal branches at PP levels > 0.90, > 0.95, and > 0.99, respectively regardless of the chosen fixed alignment. The MAP topology is also insensitive to the chosen alignment. Under the full joint model the number of supported internal branches increases to 8, 8, and 6 branches at the same levels of PP, producing an increase of 0, 1, and 2 branches.

**Figure 6:**
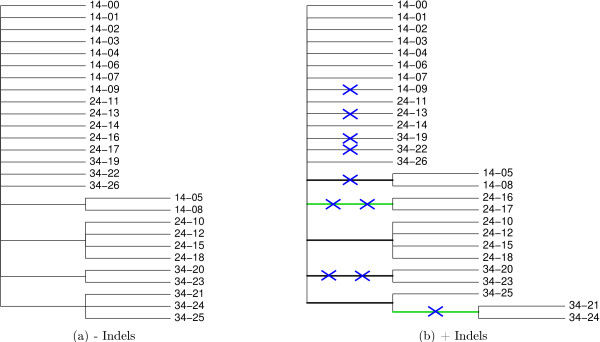
**Triplet alignments may shift indels and cause misaligned residues**. (a) At posterior probability > 0.99 the traditional sequential model supports 4 internal branches. (b) When indel information is included, the number of supported branches increases to 6. Branches colored green are supported only when indel information is incorporated. Each blue cross denotes an indel event occurring on a particular branch. Prefixes on sequence names indicate elapsed time in weeks between the initial infection and when the sequences were obtained.

While the number of branches supported at PP > 0.9 is equal, not all supported branches are the same. The number of branches supported only under the joint model is 2, 2, and 2. The joint model supports the clades (16,17) and (21,24) over the traditional model at all three levels of PP. The traditional model supports the clade (19,21,24,25) at a PP of 0.980 compared to 0.887 with indel information. The traditional model also supports the clade (10,12,15,16,18,19,21,24,25) at PP > 0.9 that has support < 0.5 when indel information is included. This results because the large clade conflicts with the clade (16,17) that is supported by two shared indels. Thus, the number of branches supported in only one of the two models at each level of PP is 4, 3, and 2. Since the joint model balances substitution and indel information as well as taking alignment ambiguity into account we assume that these differences represent an improvement in the accuracy of estimation. However, because the true tree is not observed, we cannot be certain which, if any, of the predictions is correct. The partitions supported under the two models at PP > 0.99 are depicted in Figure 6. In summary, indel information conflicts with one branch in the substitution-only tree and down-weights the evidence for another branch. The conflicting branch is ruled out by the support of 2 shared indels for the clade (16,17), although one of these is homoplastic.

### MCMC Improvements

We demonstrate that the novel MCMC transition kernel introduced in the sub-section *Sampling *improves the computational efficiency of topology estimation when using indel information. The transition kernel improves the convergence properties of the Markov chain substantially, so that fewer initial samples must be discarded as "burn-in". We compare the behavior of the estimation procedure when the new transition kernel is disabled (NNI-only) or enabled (NNI+SPR) by running 15 instances of each chain starting from a randomly chosen tree and alignment. We use the data-set from Example 2 that consists of 27 HIV sequences, with a maximum length 612 nucleotides.

To assess convergence for each Markov chain, we count the number of iterations required for the sampled tree topology to approach its equilibrium distribution of tree topologies. To accomplish this task, we need to define a distance from a single tree topology to a distibution of tree topologies. We start with the Robinson-Foulds distance (RF) between two tree topologies that we denote as *d*_RF_(*τ*_1_, *τ*_2_). This distance does not depend on branch lengths. We then define the distance *d*(*τ*_1_, *ξ*) from a topology *τ*_1 _to a distribution of topologies *ξ *as the average RF distance between *τ*_1 _and a tree *τ*_2 _~ *ξ*:

*d*(*τ*_1_, *ξ*) = E{*d*_RF_(*τ*_1_, *τ*_2_)}.     (10)

The expectation of *d*(*τ*_1_, *ξ*) does not converge to 0 as the Markov chain approaches stationarity; rather the expectation approaches the average distance between two trees sampled from the equilibrium topology distribution. With this in mind, we consider a chain to have converged when the distance from the chain's current topology to the equilibrium distribution reaches the lower 25th percentile of distances from trees at stationarity to the equilibrium distribution. We approximate the equilibrium topology distribution with 200 topologies sampled at widely spaced intervals from a long-running MCMC analysis. We find that this distribution is not sensitive to the starting point of the Markov chain, and does not change when the new transition kernel is enabled.

**Figure 7:**
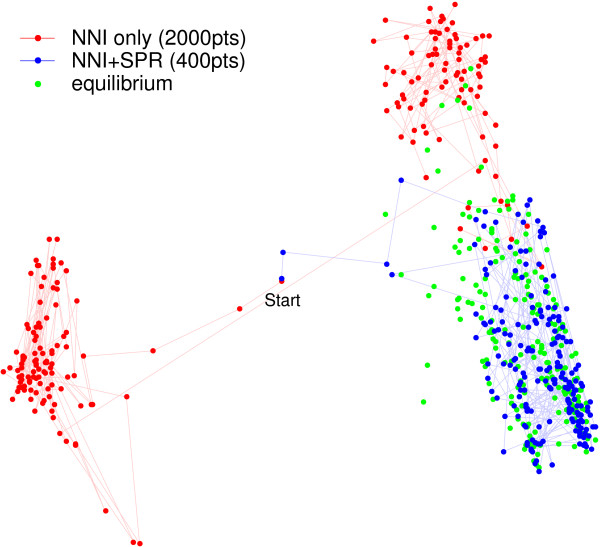
**Alignment-aware SPR transition kernel decreases burn-in time**. We consider the 27-sequence data set of HIV sequences described in the Results section as Example 2. Points represent 200 topologies sampled from a Markov chains with the alignment-aware SPR transition kernel disabled (red; NNI-only) or enabled (blue; NNI+SPR) or from the equilibrium distribution (green). While the convergence time for Markov chains varies widely, this example illustrates the median convergence time. The NNI-only chain takes 2112 iterations to converge versus only 66 iterations for the NNI+SPR chain. Because the convergence times are so different, the figure depicts every 10th tree for the first 2000 iterations, whereas for the NNI+SPR chain the figure depicts every 2nd tree for the first 400 iterations. Points represent trees projected onto the plane using multidimensional scaling based on the Robinson-Foulds distance. This distance depends only on the topology, not the branch lengths.

Without the new transition kernel based on SPR, the median time to convergence is 2112 iterations with an average of 1976.9. However, when the new transition kernel is enabled, the median time decreases to 66 iterations, and the average shrinks to 108.8 iterations. In addition, without the SPR transition kernel, the two slowest converging chains take 3887 and 6782 iterations to converge, whereas with the SPR transition kernel the two slowest converging chains require only 248 and 422 iterations to converge. Based on the average time until convergence, we calculate that the SPR transition kernel results in a roughly 18.1-fold increase in convergence speed, although we emphasize that the convergence times vary substantially around their average. The faster-converging chains spend about 2 × as much CPU time per iteration, leading to an effective speed-up of about 9-fold. To visualize the convergence properties of the two approaches, we project the tree samples from two typical chains into the plane using metric multidimensional scaling based on their RF distances (Figure 7).

## Discussion

Some researchers question the ability of indel information to improve phylogenetic resolution of recently diverged taxa. Golenberg et al. analyze non-coding spacer regions between chloroplast genes in a parsimony framework and claim that indels shared by state recur more often than substitutions shared by state [32], leading to a concern that indels are not reliable characters for phylogenetic analysis. However, Simmons and Ochoterana find indels to be reliable markers with low levels of homoplasy [33]. This contrast is partially explained by noting that the original Golenberg study incorrectly codes overlapping gaps of different lengths as homologous, leading to false homoplasy. Improved methods of coding indels when gaps overlap can lead to more accurate and more informative indel characters [33,34]. In addition, researchers note that chloroplast intergenic spacers contain indel "hotspots" and that sequence duplications or changes in the number of tandem repeats occur at a significantly higher rate than non-repeat indels [32,10]. This high rate can lead to identical but non-homologous insertions in different taxa, and so repeat indels experience higher homoplasy than non-repeat indels [9]. Repeat indels should therefore be down-weighted, but unfortunately an appropriate weighting scheme has not yet been developed [35]. We also note that current alignment algorithms do not recognize duplications or indel hotspots, so that automatic alignments must be adjusted manually. Despite these difficulties with repeat indels, researchers have examined intergenic spacers in various plant species using improved indel coding and find that indel information is consistent with substitution information and largely reinforces it, improving phylogenetic resolution and support [9,10]. In some analyses, indels are useful only in distinguishing larger groups [36]. Despite the utility of indels in phylogeny estimation, most researchers note difficulties in indel coding that result from alignment ambiguity [35]. This can be true even when the number of substitutions is too small to yield well-resolved phylogenies. While alignment ambiguity causes general problems with gap placement, some specific problems are worthy of mention. For example, aligning insertions of questionable homology may create spurious evidence for common ancestry [35]. Also, when the number of tandem sequence repeats decreases, it is unclear which repeat has been deleted. Resolving these ambiguities to yield a single alignment can increase the support for some trees while decreasing the support for others, leading to bias, and so regions whose homology is uncertain should be thrown out [35]. The joint estimation approach we advocate sidesteps many of the issues through the assignment of uncertainty on alignments, indel existence and placement.

Although the indel model described here improves on common multiple alignment algorithms by allowing indels to be shared by descent, it has some limitations. First, the model assumes that the indel rate is spatially homogeneous. However, biological sequences contain indel "hotspots" where indels are more likely to occur as well as invariant regions where indels are prohibited. Incorrectly accounting for rates at which indels occur in different regions can lead to over-weighting of the indel evidence. Clustal W attempts to place indels in hydrophilic regions of amino acid sequences, but does not have a mechanism for locating hotspots in non-coding sequences or hotspots resulting from weak selection or positive selection. Second, the indel model makes the common assumption that residues in a single sequence are never homologous. Duplications violate this assumption and are treated as insertions of random sequence by the indel process. Third, changes in the number of tandem repeats of a short sequence often occur at a higher rate than other indels via slipped-strand mispairing (SSM). However, no commonly used alignment program accounts for within-sequence homology or SSM. An improved stochastic process model that accounts for these properties of biological sequences is highly desirable in order to accurately weight shared indel evidence and to produce both more accurate alignments and phylogenies.

## Conclusion

We extend the joint Bayesian estimation framework of Redelings and Suchard [24] for recently diverged SIV and HIV sequences to incorporate indel information into phylogeny estimates. In both examples, the use of indel information increases the number of supported bi-partitions even though the branch lengths are small, especially at high posterior probabilities. While many indels in these data sets occur in a single taxon or on a branch supported by many substitutions, some indels occur on branches with few or no substitutions. The relative weight of indels and substitutions shared by descent is specified by the relative rate *λ *estimated from the data. This offers an improvement over existing methods that force the relative weight to be set a priori.

Alignment uncertainty is significant in the SIV data set. This uncertainty is illustrated by the fact that the topology distribution under the traditional model varies significantly depending on the choice of alignment (Table 1). The joint estimation framework does not suffer from this sensitivity to alignment choice and allows alignment uncertainty to be estimated (Figure 4). We note that including indel information in analyses exaggerates the bias that results from fixing a single alignment choice. The high level of alignment uncertainty in the SIV data set is partially explained by a large number of occurrences of the triplet CAA. We note that in the HIV data set alignment uncertainty does not significantly effect the topology posterior.

Models such as M0 assume that codons are unbreakable, but the HIV data set shows that this can be unrealistic. Forcing indels to codon boundaries results in a decrease in model fit of 24.1 log units because of an increase in the number of inferred substitutions. Thus choosing a codon model over a singlet model involves a tradeoff between a substantially improved substitution model and a possibility of incorrect homology in the alignment. Because the effects of the latter can be significant when the total number of substitutions is small, we welcome the development of an improved substitution model that does not force this tradeoff. Such a substitution model would be able to calculate the likelihood of a singlet alignment while making use of codon frequencies and differentiating between synonymous and non-synonymous changes.

## Methods

### Detailed Balance for Collapsed-Point Transition Kernels

We begin by considering a probability distribution *π*(*x*) on points *x *∈ Ω and a function *f*(*x*) that associates a subset of Ω to each point *x *∈ Ω. We call *f*(*x*) a collapsing function if for any *x *and *y *in Ω we have *x *∈ *f*(*x*) and *f*(*x*) and *f*(*y*) are either identical or disjoint. If *f *is a collapsing function, then it partitions Ω into a set of non-overlapping subsets, which we refer to as collapsed points. We denote the set of collapsed points as *f*(Ω), and note that the probability π*(*f*(*x*)) of each collapsed point *f*(*x*) can be naturally defined as the integral of the probabilities *π*(*y*) of points *y *∈ *f*(*x*). Because the collapsed points are disjoint sets, these probabilities sum to 1 and yield a probability distribution on collapsed points.

We then consider a transition kernel *P *on Ω that is defined in terms of a transition kernel P* on *f*(Ω). Starting from the current point *x*, this transition kernel consists of collapsing *x *to *f*(*x*), moving to some other collapsed point *a*, and then selecting a point *y *from *a *in proportion to its probability *π*(*y*). We note that *y *∈ *a *implies *a *= *f*(*y*) and write the probability expression for this transition kernel as

P(x,y)=P∗(f(x),f(y))×π(y)π∗(f(y)).     (11)
 MathType@MTEF@5@5@+=feaafiart1ev1aaatCvAUfKttLearuWrP9MDH5MBPbIqV92AaeXatLxBI9gBaebbnrfifHhDYfgasaacH8akY=wiFfYdH8Gipec8Eeeu0xXdbba9frFj0=OqFfea0dXdd9vqai=hGuQ8kuc9pgc9s8qqaq=dirpe0xb9q8qiLsFr0=vr0=vr0dc8meaabaqaciaacaGaaeqabaqabeGadaaakeaacqWGqbaucqGGOaakcqWG4baEcqGGSaalcqWG5bqEcqGGPaqkcqGH9aqpcqWGqbaudaahaaWcbeqaaiabgEHiQaaakiabcIcaOiabdAgaMjabcIcaOiabdIha4jabcMcaPiabcYcaSiabdAgaMjabcIcaOiabdMha5jabcMcaPiabcMcaPiabgEna0oaalaaabaacciGae8hWdaNaeiikaGIaemyEaKNaeiykaKcabaGae8hWda3aaWbaaSqabeaacqGHxiIkaaGccqGGOaakcqWGMbGzcqGGOaakcqWG5bqEcqGGPaqkcqGGPaqkaaGaeiOla4IaaCzcaiaaxMaadaqadaqaaiabigdaXiabigdaXaGaayjkaiaawMcaaaaa@57FF@

The condition for *P *to satisfy detailed balance is

π(x)×P∗(f(x),f(y))π(y)π∗(f(y))=π(y)×P∗(f(y),f(x))π(x)π∗(f(x)).     (12)
 MathType@MTEF@5@5@+=feaafiart1ev1aaatCvAUfKttLearuWrP9MDH5MBPbIqV92AaeXatLxBI9gBaebbnrfifHhDYfgasaacH8akY=wiFfYdH8Gipec8Eeeu0xXdbba9frFj0=OqFfea0dXdd9vqai=hGuQ8kuc9pgc9s8qqaq=dirpe0xb9q8qiLsFr0=vr0=vr0dc8meaabaqaciaacaGaaeqabaqabeGadaaakeaaiiGacqWFapaCcqGGOaakcqWG4baEcqGGPaqkcqGHxdaTcqWGqbaudaahaaWcbeqaaiabgEHiQaaakiabcIcaOiabdAgaMjabcIcaOiabdIha4jabcMcaPiabcYcaSiabdAgaMjabcIcaOiabdMha5jabcMcaPiabcMcaPmaalaaabaGae8hWdaNaeiikaGIaemyEaKNaeiykaKcabaGae8hWda3aaWbaaSqabeaacqGHxiIkaaGccqGGOaakcqWGMbGzcqGGOaakcqWG5bqEcqGGPaqkcqGGPaqkaaGaeyypa0Jae8hWdaNaeiikaGIaemyEaKNaeiykaKIaey41aqRaemiuaa1aaWbaaSqabeaacqGHxiIkaaGccqGGOaakcqWGMbGzcqGGOaakcqWG5bqEcqGGPaqkcqGGSaalcqWGMbGzcqGGOaakcqWG4baEcqGGPaqkcqGGPaqkdaWcaaqaaiab=b8aWjabcIcaOiabdIha4jabcMcaPaqaaiab=b8aWnaaCaaaleqabaGaey4fIOcaaOGaeiikaGIaemOzayMaeiikaGIaemiEaGNaeiykaKIaeiykaKcaaiabc6caUiaaxMaacaWLjaWaaeWaaeaacqaIXaqmcqaIYaGmaiaawIcacaGLPaaaaaa@7917@

By cancelling common terms.

π* (*f*(*x*)) × P*(*f*(*x*), *f*(*y*)) = π* (*f*(*y*)) × P*(*f*(*y*), *f*(*x*)).     (13)

Thus, the requirement for *P *to satisfy detailed balance on Ω is simply that P* satisfies detailed balance on *f*(Ω).

We now demonstrate that the function *f *that maps (**A**, *τ*, **T**, **Θ**, **Λ**) to (∗,τ,T,Θ,Λ)C2(A,τ,PO)
 MathType@MTEF@5@5@+=feaafiart1ev1aaatCvAUfKttLearuWrP9MDH5MBPbIqV92AaeXatLxBI9gBaebbnrfifHhDYfgasaacH8akY=wiFfYdH8Gipec8Eeeu0xXdbba9frFj0=OqFfea0dXdd9vqai=hGuQ8kuc9pgc9s8qqaq=dirpe0xb9q8qiLsFr0=vr0=vr0dc8meaabaqaciaacaGaaeqabaqabeGadaaakeaacqGGOaakcqGHxiIkcqGGSaaliiGacqWFepaDcqGGSaalieqacqGFubavcqGGSaaliiqacqqFyoqucqGGSaalcqqFBoatcqGGPaqkdaWgaaWcbaGaem4qam0aaSbaaWqaaiabikdaYaqabaWccqGGOaakcqWGbbqqcqGGSaalcqWFepaDcqGGSaalcqqGqbaucqqGpbWtcqGGPaqkaeqaaaaa@43A4@ is a collapsing function. The directed branch PO partitions the nodes of *τ *into two subsets excluding node O (Figure 2). Set *C*_2 _contains all alignments that are consistent with **A **on each of the two subsets. Alignment **A **certainly fulfills this criterion, and therefore **A **∈ *C*_2_(**A**, *τ*, PO), implying that *x *∈ *f*(*x*) for any *x*. In addition, *C*_2_(**A***'*, *τ*, PO) = *C*_2_(**A**, *τ*, PO) for any **A***' *in *C*_2_(**A**, *τ*, PO) and so *f*(*y*) = *f*(*x*) for any *y *∈ *f*(*x*), implying that *f*(*x*) and *f*(*y*) are either identical or non-overlapping. Therefore *f*(*x*) is a collapsing function. The transition kernel consisting of SPR proposals for points collapsed using *C*_2_(**A**, *τ*, PO) therefore satisfies detailed balance when we use the MH rule for acceptance or rejection and MH satisfies detailed balance on the collapsed points.

### Collapsed Sampling as an MH Proposal Distribution

Our method for sampling alignments samples from a distribution *η *that approximates the correct distribution *π *but does not match exactly [24]. We therefore define an MH transition kernel that uses collapsed sampling of alignments as a proposal distribution *ρ*. After selecting a new topology and alignment that goes along with it, we reject this new point *j *and move back to the original alignment and topology *i *with a small probability 1 - *α*_*ij*_. The MH acceptance ratio can be calculated as follows:

πiρijαij=πjρjiαji,αijαji=πjρjiπiρij.     (14)
 MathType@MTEF@5@5@+=feaafiart1ev1aaatCvAUfKttLearuWrP9MDH5MBPbIqV92AaeXatLxBI9gBaebbnrfifHhDYfgasaacH8akY=wiFfYdH8Gipec8Eeeu0xXdbba9frFj0=OqFfea0dXdd9vqai=hGuQ8kuc9pgc9s8qqaq=dirpe0xb9q8qiLsFr0=vr0=vr0dc8meaabaqaciaacaGaaeqabaqabeGadaaakeaafaqaaeGadaaabaacciGae8hWda3aaSbaaSqaaiabdMgaPbqabaGccqWFbpGCdaWgaaWcbaGaemyAaKMaemOAaOgabeaakiab=f7aHnaaBaaaleaacqWGPbqAcqWGQbGAaeqaaaGcbaGaeyypa0dabaGae8hWda3aaSbaaSqaaiabdQgaQbqabaGccqWFbpGCdaWgaaWcbaGaemOAaOMaemyAaKgabeaakiab=f7aHnaaBaaaleaacqWGQbGAcqWGPbqAaeqaaOGaeiilaWcabaWaaSaaaeaacqWFXoqydaWgaaWcbaGaemyAaKMaemOAaOgabeaaaOqaaiab=f7aHnaaBaaaleaacqWGQbGAcqWGPbqAaeqaaaaaaOqaaiabg2da9aqaamaalaaabaGae8hWda3aaSbaaSqaaiabdQgaQbqabaGccqWFbpGCdaWgaaWcbaGaemOAaOMaemyAaKgabeaaaOqaaiab=b8aWnaaBaaaleaacqWGPbqAaeqaaOGae8xWdi3aaSbaaSqaaiabdMgaPjabdQgaQbqabaaaaOGaeiOla4caaiaaxMaacaWLjaWaaeWaaeaacqaIXaqmcqaI0aanaiaawIcacaGLPaaaaaa@6759@

The *ρ*_*ij *_satisfy detailed balance with respect to another probability *η*_*i *_= *π*_*i*_*f*_*i*_. Thus,

ηiρij=ηjρjiπifiρij=πjfjρjififj=πjρjiπiρij.     (15)
 MathType@MTEF@5@5@+=feaafiart1ev1aaatCvAUfKttLearuWrP9MDH5MBPbIqV92AaeXatLxBI9gBaebbnrfifHhDYfgasaacH8akY=wiFfYdH8Gipec8Eeeu0xXdbba9frFj0=OqFfea0dXdd9vqai=hGuQ8kuc9pgc9s8qqaq=dirpe0xb9q8qiLsFr0=vr0=vr0dc8meaabaqaciaacaGaaeqabaqabeGadaaakeaafaqaaeWadaaabaacciGae83TdG2aaSbaaSqaaiabdMgaPbqabaGccqWFbpGCdaWgaaWcbaGaemyAaKMaemOAaOgabeaaaOqaaiabg2da9aqaaiab=D7aOnaaBaaaleaacqWGQbGAaeqaaOGae8xWdi3aaSbaaSqaaiabdQgaQjabdMgaPbqabaaakeaacqWFapaCdaWgaaWcbaGaemyAaKgabeaakiabdAgaMnaaBaaaleaacqWGPbqAaeqaaOGae8xWdi3aaSbaaSqaaiabdMgaPjabdQgaQbqabaaakeaacqGH9aqpaeaacqWFapaCdaWgaaWcbaGaemOAaOgabeaakiabdAgaMnaaBaaaleaacqWGQbGAaeqaaOGae8xWdi3aaSbaaSqaaiabdQgaQjabdMgaPbqabaaakeaadaWcaaqaaiabdAgaMnaaBaaaleaacqWGPbqAaeqaaaGcbaGaemOzay2aaSbaaSqaaiabdQgaQbqabaaaaaGcbaGaeyypa0dabaWaaSaaaeaacqWFapaCdaWgaaWcbaGaemOAaOgabeaakiab=f8aYnaaBaaaleaacqWGQbGAcqWGPbqAaeqaaaGcbaGae8hWda3aaSbaaSqaaiabdMgaPbqabaGccqWFbpGCdaWgaaWcbaGaemyAaKMaemOAaOgabeaaaaGccqGGUaGlaaGaaCzcaiaaxMaadaqadaqaaiabigdaXiabiwda1aGaayjkaiaawMcaaaaa@70C5@

Therefore the acceptance ratio is:

αijαji=fifj.     (16)
 MathType@MTEF@5@5@+=feaafiart1ev1aaatCvAUfKttLearuWrP9MDH5MBPbIqV92AaeXatLxBI9gBaebbnrfifHhDYfgasaacH8akY=wiFfYdH8Gipec8Eeeu0xXdbba9frFj0=OqFfea0dXdd9vqai=hGuQ8kuc9pgc9s8qqaq=dirpe0xb9q8qiLsFr0=vr0=vr0dc8meaabaqaciaacaGaaeqabaqabeGadaaakeaadaWcaaqaaGGaciab=f7aHnaaBaaaleaacqWGPbqAcqWGQbGAaeqaaaGcbaGae8xSde2aaSbaaSqaaiabdQgaQjabdMgaPbqabaaaaOGaeyypa0ZaaSaaaeaacqWGMbGzdaWgaaWcbaGaemyAaKgabeaaaOqaaiabdAgaMnaaBaaaleaacqWGQbGAaeqaaaaakiabc6caUiaaxMaacaWLjaWaaeWaaeaacqaIXaqmcqaI2aGnaiaawIcacaGLPaaaaaa@4257@

Distribution *f*_*i *_is proportional to the product of the length distributions on the internal nodes and changes very slowly in *i*. Therefore fifj
 MathType@MTEF@5@5@+=feaafiart1ev1aaatCvAUfKttLearuWrP9MDH5MBPbIqV92AaeXatLxBI9gBaebbnrfifHhDYfgasaacH8akY=wiFfYdH8Gipec8Eeeu0xXdbba9frFj0=OqFfea0dXdd9vqai=hGuQ8kuc9pgc9s8qqaq=dirpe0xb9q8qiLsFr0=vr0=vr0dc8meaabaqaciaacaGaaeqabaqabeGadaaakeaadaWcaaqaaiabdAgaMnaaBaaaleaacqWGPbqAaeqaaaGcbaGaemOzay2aaSbaaSqaaiabdQgaQbqabaaaaaaa@3280@ is usually quite close to one and there are few rejections.

### Assessing Alignment Ambiguity

To assess alignment ambiguity we compare the posterior topology distribution for the full joint model to the distribution generated under models restricted to a fixed alignment. As these distributions may be sensitive to the specific alignment chosen, we use three different choices. These alignments are the estimates obtained from Clustal W [37], Muscle [38], and BAli-Phy [24]. In the latter case, we fix the alignment to its Maximum A Posteriori (MAP) point determined jointly. We use the default parameters for Clustal W and Muscle. Parameters and models used by BAli-Phy are described in the *Results *section.

### Computation Time and Problem Size

The inference method described in this paper and implemented in the BAli-Phy software [24] requires significant computation time in order to handle alignment uncertainty and incorporate indel information. This means that it is often impractical to analyze data sets with greater than 12 taxa or sequence lengths longer than about 750 letters (nucleotide, amino acid, or codon). Analyzing data sets of this size often takes about a week on current hardware. However, we wish to emphasize two points. First, the long computation time is not required to make a simple estimate, but to obtain measures of confidence that are accurate enough to publish. For simple estimates or unpublished results, significantly larger data sets can be analyzed. Second, the amount of time required to analyze a data set depends not just on the size of the data set, but on various characteristics such as the level of uncertainty. For example, the second example in this paper contains 27 taxa of maximum length 612 and took about 3 weeks to analyze.

## Authors' contributions

MS formulated the problem and provided project management. BR designed the algorithms and models. BR performed the actual programming and computations. MS and BR analyzed the data. MS and BR wrote the paper. All authors read and approved the final manuscript.
